# Projecting future mortality risk of pleural mesothelioma in Italy (2020–2034): Bayesian age–period–cohort analysis over 40 years of death registration

**DOI:** 10.3389/fpubh.2025.1741506

**Published:** 2026-01-16

**Authors:** Allegra Sartore, Giorgia Stoppa, Giada Minelli, Carolina Mensi, Dario Consonni, Valerio Manno, Alessandro Marinaccio, Lucia Fazzo, Annibale Biggeri, Dolores Catelan

**Affiliations:** 1 Unit of Biostatistics, Epidemiology and Public Health, DCTVPH, University of Padova, Padova, Italy; 2 Statistical Service, Italian National Institute of Health, Rome, Italy; 3 Occupational Health Unit, Fondazione IRCCS Ca' Granda Ospedale Maggiore Policlinico, Milan, Italy; 4 Occupational and Environmental Medicine, Epidemiology and Hygiene Department, Italian Workers' Compensation Authority (INAIL), Rome, Italy; 5 Department of Environment and Health, Italian National Institute of Health, Rome, Italy

**Keywords:** asbestos exposure, Bayesian model, cancer epidemiology, epidemiological surveillance, forecasts, pleural mesothelioma

## Abstract

**Introduction:**

Italy, a major former producer, banned asbestos in 1992. A high incidence of mesothelioma, one of the asbestos legacies, is still observed due to the long latency and exposure from residual asbestos-containing materials. Future mortality forecasts at both national and subnational levels are still lacking. This work aims to project future age-stratified mortality rates (2020–2034) for pleural mesothelioma (PM) in Italy, both nationally and for each administrative region.

**Methods:**

Data on pleural cancers and PM in Italy between 1980 and 2019 were extracted from death registries, adjusted for PM misclassification in ICD-9, and aggregated in eight periods, eighteen age classes, and fifteen birth cohorts. Bayesian age-period-cohort models were implemented to generate age-specific mortality projections, stratified by sex assigned at birth.

**Results:**

Between 1980 and 2019, 33,889 people died from PM in Italy, and 19,092 more deaths are expected between 2020 and 2034. The national peak is predicted for 2020–2024, with 6,740 deaths. Age groups under 75 years have already reached the peak of mortality rates. Region-specific trends by sex and time reflect the country’s industrialization history.

**Discussion:**

These results align with the literature in predicting the timing of the mesothelioma peak and offer new insights into age-specific trends, the rate of decline, and geographical patterns. They provide valuable evidence on the heterogeneous asbestos legacy across regions, supporting targeted public health actions and health planning.

## 1 Introduction

Asbestos is a group of minerals that was extensively used in the past due to its remarkable properties, including heat resistance and durability. However, its carcinogenic effects are well recognized, causing mesothelioma, cancers of the lung, the ovary, and the larynx, in addition to non-malignant diseases, such as asbestosis and pleural plaques. Mesothelioma is a rare and aggressive cancer primarily affecting the pleura and the peritoneum (in Italy, 93.2% and 6.3%, respectively), and asbestos is the principal carcinogen associated with it (1–3).

Following the recognition of asbestos-related health hazards, many countries, starting with Nordic European nations in the early 1980s (Sweden was the first), introduced bans on its production and import. The European Union followed with a complete ban in 2005 (4, 5). Despite the implementation of these policies over three decades ago, asbestos-related diseases (ARDs) remain a significant global burden due to the persistence of asbestos in existing structures, together with the long latency periods of diseases like mesothelioma, which can take up to many decades from first exposure to manifest. In Italy, where the asbestos ban was introduced in 1992 (Law 257/92), approximately 4,000 asbestos-related deaths per year were still recorded between 2010 and 2016, with around 1,000 of these attributed to mesothelioma (6). This proportion is consistent with findings reported in (7), indicating that mesothelioma accounts for less than 50% of all asbestos-related deaths, as lung cancer is more frequent. While a decline in mortality by birth cohort has been detected, only a slight regression in trends by calendar period has been observed so far (44).

Several studies have analyzed mesothelioma incidence or mortality trends in our country, including predictions for future mortality, and, in some cases, have also linked the Italian asbestos consumption curves with mesothelioma mortality (8–14). However, most existing works focus on national data, primarily on the male population, based on the past consumption of asbestos, and only three have addressed single local or regional data (13–15).

The present study aims to produce age-stratified projections of mortality from pleural mesothelioma (PM) in Italy, using 40 years of death registration data, both at national and regional levels, separately by sex assigned at birth.

## 2 Materials and methods

Data on deaths caused by pleural malignant tumors (ICD-9: 163) and PM (ICD-10: C45.0) were extracted by the Statistical Service of the Italian National Institute of Health from the National Mortality Database of the National Census Bureau (ISTAT) for the period 1980–2019, both at the national and regional levels and separately for males and females. Unfortunately, the ICD-9 code 163 does not distinguish pleural mesothelioma from other pleural tumors. To correct for this misclassification, we utilized the multiple cause of death registration data from 1995 to 2002, as described in detail in the following section.

### 2.1 Correction for ICD-9 misclassification

For the period from 1995 (the year the multiple cause of death registries became available) to 2002 (the last year the ICD-9 classification was in force), we used two sources of data: the multiple cause of death registries and the National Mortality Database, both maintained by ISTAT. The multiple cause of death registries are single anonymous electronic registries that contain all four causes of death exactly as typed in full by the coroner on the death certificate (underlying, intermediate, terminal, and accompanying cause). To isolate pleural mesothelioma from other pleural tumors, we first accept the four-digit codes 163.0 and 163.1 (parietal and visceral pleural tumors) as pleural mesotheliomas. Then, we considered the multiple cause of death records of people who died with code 163.9, “Malignant neoplasm of pleura, unspecified.” For them, we examined alphanumeric descriptions (clear entries) in the database of multiple causes of death. We extracted all people for whom the terms ‘mesothelioma’ and ‘pleura’ were specified as the underlying cause (i.e., the disease or injury that initiated the chain of events leading directly to death). Overall, we identified 6,242 people who died from mesothelioma of the pleura (see Table 1). We calculated these frequencies annually from 1995 to 2002—when ICD-10 became the standard—and to adjust for the years before 1995, back to 1980, we used the proportion of mesothelioma out of the total number of pleural cancers observed in 1995. Mortality and population data were extracted for each Italian administrative region. For this analysis, we aggregated the records of the two autonomous provinces of Trento and Bolzano into a single administrative area (Trentino-Alto Adige), resulting in a total of 20 regions.

**Table 1:** Pleural mesothelioma death counts and projections present in the literature, in comparison with our data (before and after the correction for ICD9 overestimation) and projections.

Calendar period.	Marinaccio et al. (10) (males)	Marinaccio et al. (10) projections (males)	Oddone et al. (8) (males)	Oddone et al. (8) projections (males)	Oddone et al. (8) (females)	Oddone et al. (8) projections (females)	Oddone et al. (9) projections (males)	Raw data (males)	Corrected data (males)	Projections (males)	Raw data (females)	Corrected data (females)	Projections (females)
1970–1974	1,218		856		500								
1975–1979	1,387		954		517								
1980–1984	1,911		1,314		676			1,926	1,332	1,334	1,111	793	825
1985–1989	2,353		1,605		794			2,379	1,639	1,641	1,353	961	956
1990–1994	2,966		2,016		926			2,998	2,042	2,047	1,645	1,146	1,105
1995–1999	3,313		2,399		1,002			3,343	2,421	2,514	1,615	1,204	1,234
2000–2004		3,000–4,100	3,163		1,286			3,749	3,224	3,103	1,662	1,451	1,382
2005–2009		3,500–4,800	3,663		1,392			3,729	3,729	3,727	1,451	1,451	1,507
2010–2014		4,000–5,500	4,275		1,569		4,275	4,352	4,352	4,336	1,634	1,634	1,652
2015–2019		4,250–5,800		4,965		1,740	5,000	5,641	4,732	4,781	2,114	1,778	1,766
2020–2024		4,500–6,200		5,331		1,815	5,600			4,946			1,794
2025–2029		4,100–5,600		5,331		1,783	5,000			4,783			1,718
2030–2034		3,600–4,900		4,928		1,654	3,500			4,279			1,571
2035–2039				4,288		1,438							

We subsequently aggregated the data into eighteen 5-year age classes (0–4 years old, …, 85+ years old) and eight 5-year calendar periods (1980–1984, …, 2015–2019). We generated fifteen birth cohorts (1905–1914, …, 1975–1984). These cohorts, highlighted in grey on the Lexis diagram, span 10 years each and partially overlap (Supplementary Table S1).

Projected future mortality rates and counts were computed conditional on ISTAT annual population projections for 2020–2034, aggregated into three 5-year calendar periods (2020–2024, 2025–2029, 2030–2034) (16).

### 2.2 Age-Period-Cohort models

A Bayesian Age-Period-Cohort (BAPC) model was specified to project future age-specific mortality rates for the period 2020–2034. All the analyses were conducted separately for males and females at the national and regional levels. The observed number of deaths $O_{\mathrm{ij}}$ , with *i* = 1, …, 18 age classes and *j* = 1, …,8 periods, is assumed to follow a Poisson distribution with parameter $n_{\mathrm{ij}}\lambda_{\mathrm{ij}}$ , where $n_{\mathrm{ij}}$ is the known person-years at risk. The APC model specifies a log-linear model for the rates:


$$\log(\lambda_{\mathrm{ij}})=\alpha +\vartheta_{i}+\mu_{j}+\nu_{k}$$

Where $\theta_{i}$ , $\mu_{j}$ and $\nu_{k}$ are age, period, and cohort effects (*k* = 1, …, 25), respectively (17). Suppose we want to project mortality for the future *t*-th periods ahead for a given *i*-th age class; the model becomes:


$$\log(\lambda_{i,J+t})=\alpha +\vartheta_{i}+\mu_{J+t}+\nu_{k(i,J+t)}$$

Since birth cohort terms—running on the diagonals of the Lexis diagram—are linearly defined by age and period. Note that for future predictions, most cohort terms do not need to be predicted. In the Bayesian specification, age, period, and cohort effects are assumed *a priori* to follow a second-order random walk (RW2), reflecting the idea that adjacent time points tend to exhibit similar risk patterns, without enforcing a rigid, constant trend. We acknowledge that using a RW2 prior could introduce the risk of oversmoothing, but we preferred it over RW1. Moreover, under this assumption, identifiability is granted since second-order differences are implied, and we are interested in the log-linear predictors, not in the individual quantities. Additional heterogeneity terms are also considered to account for overdispersion. Finally, predictions are based on predictive distributions derived from Poisson distributions with an expected value given by the predicted log-linear predictor and the ISTAT annual population projections for 2020–2034. Further details in (18).

We used the R package BAPC with Integrated Nested Laplace Approximations (INLA) (18). This approach enables the approximation of posterior marginal distributions for future mortality counts, thereby avoiding the need for MCMC sampling techniques. All statistical analyses were conducted using R version 4.4.2 (R Core Team 2024).

The age-standardised death rates were calculated using the WHO World Standard Population (19). Age-standardised rates based on these weights will privilege younger-adult age classes and will show a peak earlier than other weighting schemes. As a comparison, we also provide age-standardised death rates according to the 2013 revision of the European Standard Population—which has a slightly older age structure—in the Supplementary material. Using the absolute number of deaths is equivalent to weighting according to the number of deaths by age class, therefore giving more importance to the age classes with higher age-specific rates—i.e., the older age classes. Then, using absolute numbers will show a peak in later or future calendar periods.

### 2.3 Predictive quality assessment

To assess the predictive quality of our projections, a continuous rank probability score (CRPS) was calculated for the years in which both predicted and observed values were available, as reported in Riebler and Held’s work (18). It measures the sharpness and calibration of forecasts, hence the lower the better. For predictions with zero variance, CRPS is equal to the Absolute Error. An unconditional calibration test based on the mean value of CRPS, with the null hypothesis of perfect calibration, was then conducted [refer to (18) for computational details].

## 3 Results

Between 1980 and 2019, 33,889 deaths from PM occurred in Italy (23,471 males and 10,418 females). A total of 19,092 more deaths [14,008 (90% CrI: 13,555, 14,463) males and 5,084 (90% CrI: 4,799, 5,371) females] are expected to occur between 2020 and 2034. The absolute peak is predicted for 2020–2024, with 6,740 deaths [4,946 (90% CrI: 47,57, 5,136) males and 1,794 (90% CrI: 1,687, 1,903) females]. Following the peak, 6,501 deaths are expected in 2025–2029 [4,783 (90% CrI: 4,528, 5,039) males and 1,718 (90% CrI: 1,563, 1,873) females], and 5,850 in 2030–2034 [4,279 (90% CrI: 3,955, 4,603) males and 1,571 (90% CrI: 1,356, 1,787) females].

Table 1 shows the projections of PM mortality in Italy extracted from existing literature (8–10), stratified by sex where available, compared to our results. Specifically, we reported our data both raw (i.e., with no ICD-9 correction) and corrected for the misclassification, in addition to the model projections, all stratified by sex.

Our estimates are lower than the previous ones. Supplementary Table S2 reports the M/F ratios (observed and predicted) calculated from these data.

**Figure 1:**
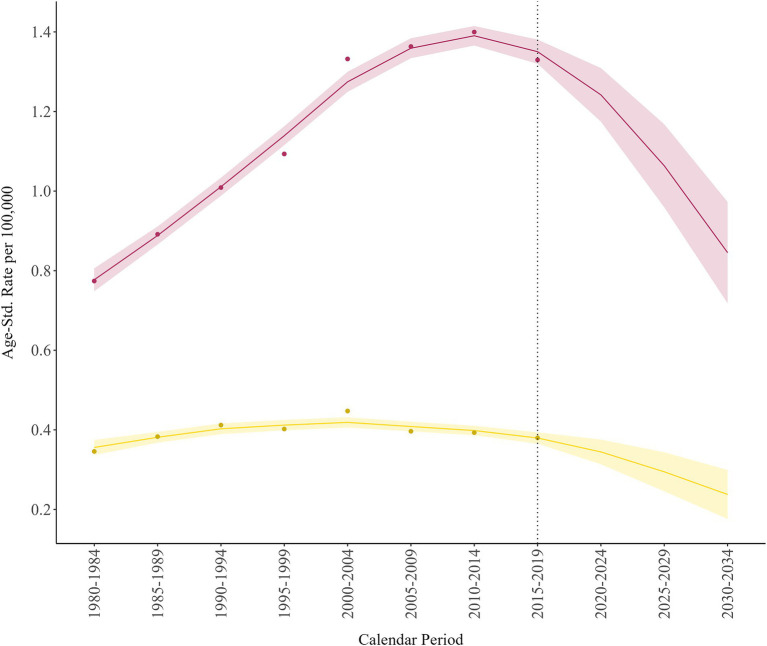
Pleural mesothelioma projected and observed (dots) age-standardised mortality rates, for males (dark red) and females (sand), with 90% credibility intervals. Italy, 1980–2034.

**Figure 2:**
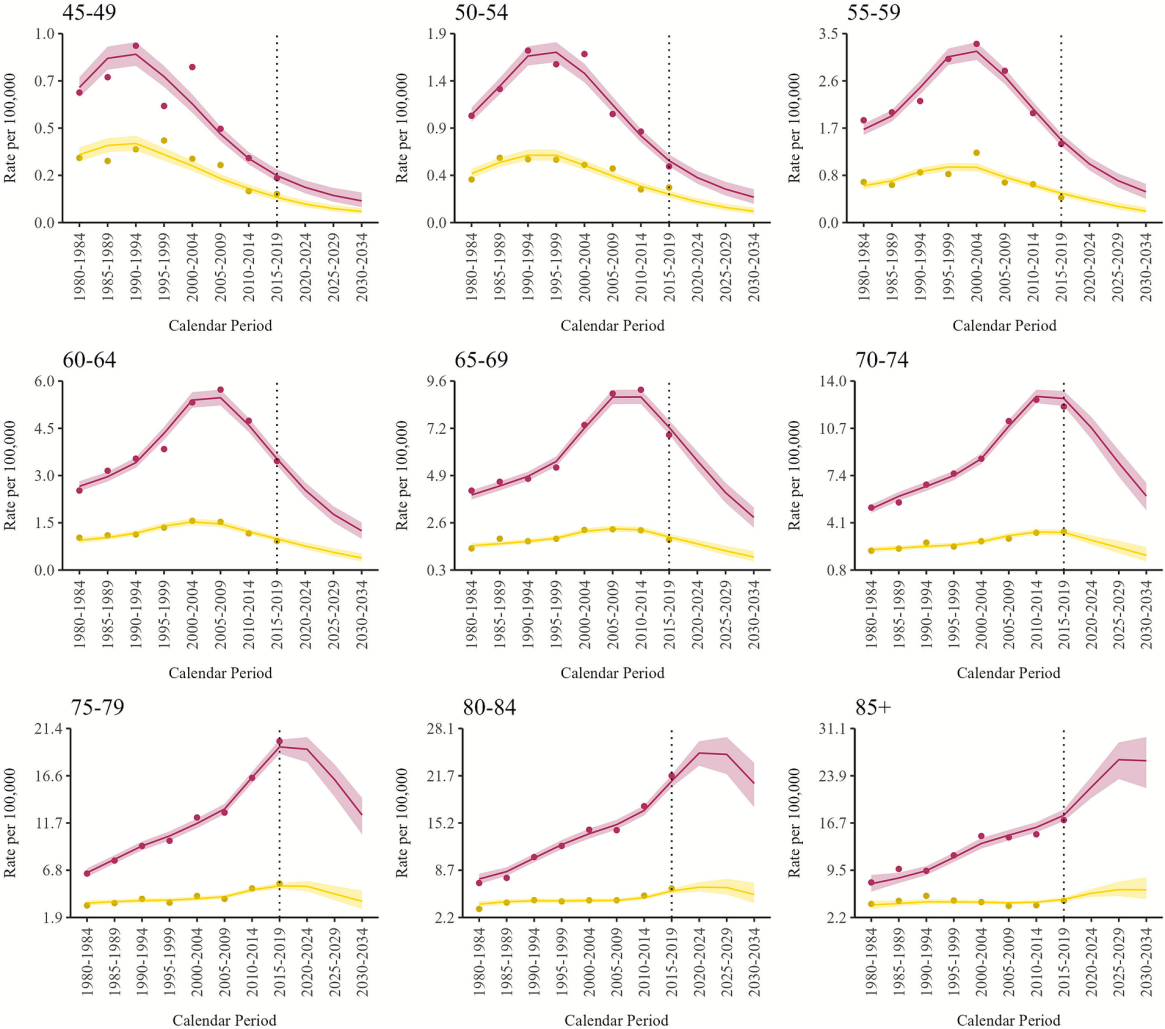
Pleural mesothelioma projected and observed (dots) age-specific mortality rates by calendar period, for males (dark red) and females (sand) with 90% credibility intervals. Italy, 1980–2034.

Figure 1 shows the projected (and observed as dots) national age-standardised death rates (according to the WHO World Standard Population) per 100,000, for the two sexes, together with their 90% credibility intervals. The dotted vertical line represents the start of the predicted rates. These estimates encompass all age classes and place the age-standardised peak in the period 2010–2014 for males [1.390 (90% CrI: 1.366, 1.415) per 100,000] and 2000–2004 for females [0.419 (90% CrI: 0.406, 0.432) per 100,000], approximately 10–20 years earlier than the absolute peak. The peak in terms of standardised rates already occurred, confirming a decreasing trend in PM in Italy. Standardised death rates according to the 2013 revision of the European Standard Population (Supplementary Figure S1) showed identical trends across calendar periods, but with higher rate values, because this standard population is older than the WHO standard population. However, the mesothelioma mortality peaks are predicted at different calendar periods by age class. Only after the age of 75, the peaks are predicted in future calendar periods (2024+). Therefore, any average over age classes could depend on the age-specific weights used. For the reasons already explained in the previous section, we recommend that the readers focus on the age-specific patterns shown in Figure 2.

Figure 2 illustrates the national age-specific mortality rates for males and females by calendar period, as projected by the BAPC models, along with their 90% credibility intervals. The observed rates are marked as dots. Only age classes starting from the 45–49 one are included, as younger groups experience a very low mortality, leading to imprecise estimates with wider credibility bounds. The projections for the male population indicate a significant decline across nearly all age classes, except the oldest age group, which reaches a plateau in the last projected period. A similar trend is evident in the projections for females, although only up to 75 years of age. Beyond this age, the projected decline becomes less distinct, and the credibility intervals widen. The mortality peaks are coherent between males and females: age class 45–49 shows a peak in 1990–1994; age class 55–59 in 2000–2004; age class 65–69 in 2010–2014; age class 75–79 in 2015–2019, and age class 85+ in 2025–2029. In other words, peaks are coherent with the highest risk shown for the birth cohorts born between 1940 and 1954.

**Figure 3:**
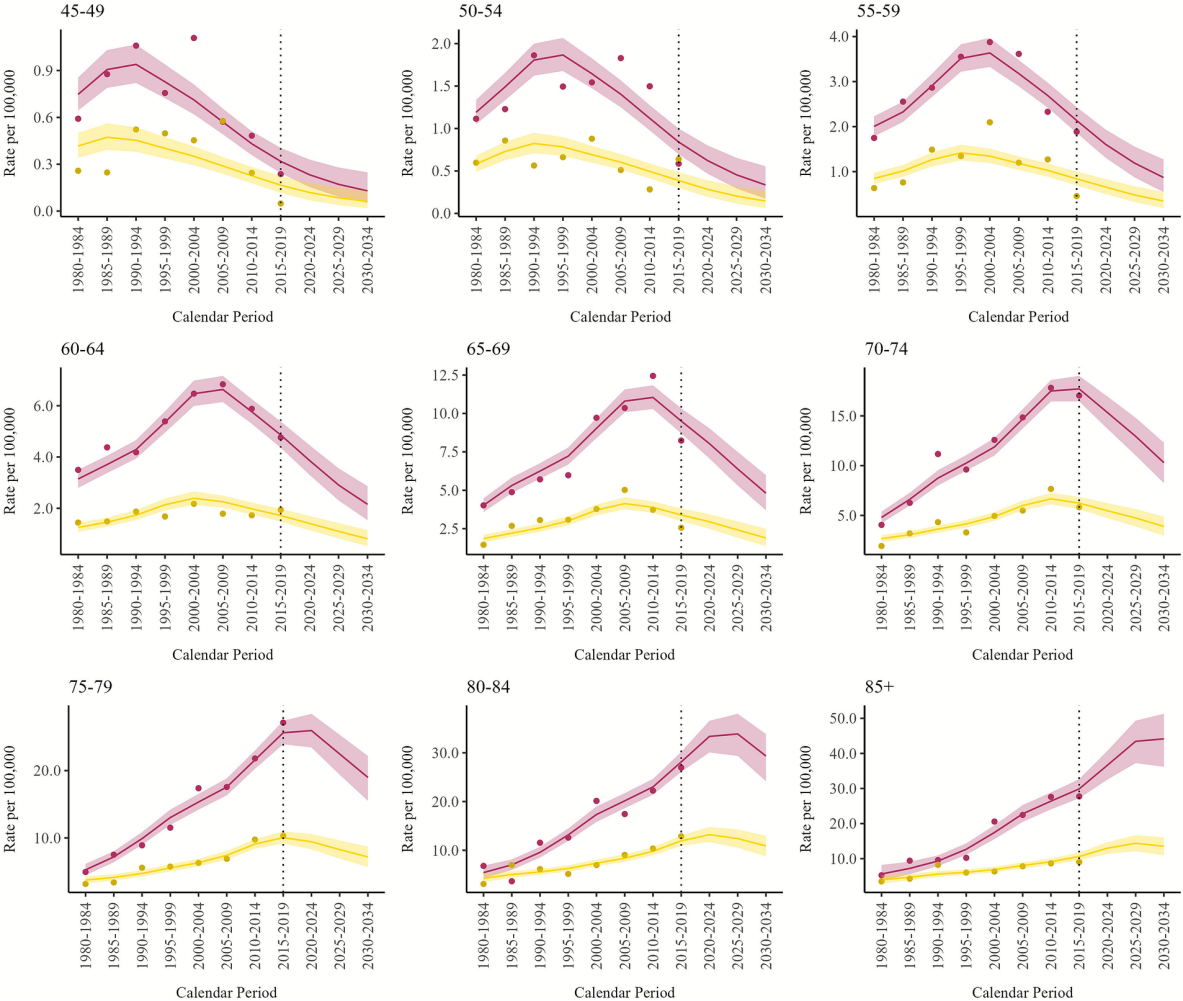
Pleural mesothelioma projected and observed (dots) age-specific mortality rates (per 100,000) by calendar period, for males (dark red) and females (sand) with 90% credibility intervals. Lombardy, 1980–2034.

**Figure 4:**
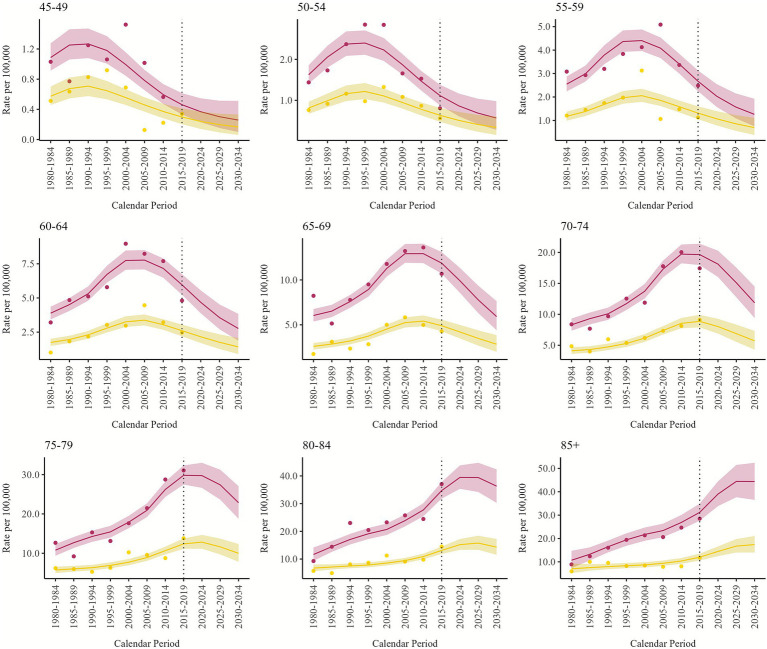
Pleural mesothelioma projected and observed (dots) age-specific mortality rates (per 100,000) by calendar period, for males (dark red) and females (sand) with 90% credibility intervals. Piedmont, 1980–2034.

Projections were also made for each Italian administrative region. We included all the regional profiles in Supplementary Figures S2–S19, although here we focus on the results for the four regions most affected by asbestos exposure: Lombardy, Piedmont (Figures 3, 4), Friuli Venezia Giulia, and Liguria (Supplementary Figures S7, S9). Regional predictions have wider credibility intervals because the lower number of deaths makes the models more unstable and the estimated rates less precise. The predicted trends are similar to the national ones, with a sharp decrease observed in the age group 65–69 for both males and females. Among males, in Lombardy, the trend in the 70–74 age group is still stable in 2015–2019, while in Piedmont and Friuli Venezia Giulia we observe a decrease since 2015–2019, and in Liguria the peak was observed in 2005–2009. Among females, Lombardy showed a decrease in the 70–74 age group since 2010–2014, while in Piedmont it is still increasing in 2015–2019.

We conducted a calibration test, at the national level, using the age-specific CRPS scores averaged over all periods. The corresponding *z*-statistics and *p*-values (in parentheses) are reported in Supplementary Table S3. No age class shows signs of miscalibration, according to this test.

### 3.1 Sensitivity analysis: aggregated or single-year projections

We computed predictions using data aggregated in the Lexis diagram by 5-year classes, both in age and calendar period dimensions. Riebler and Held (18) used 1-year age classes with a non-symmetric Lexis diagram. We implemented, as a sensitivity analysis, the APC model also on 1-year age class data. The results are consistent even if the predictions are wiggly (data not shown). This sensitivity analysis suggests a peak for males starting by 2017 and for females by 2023.

## 4 Discussion

In this study, we estimate mortality projections due to PM in Italy until 2034, predicting an absolute peak of around seven thousand deaths in 2020–2024, followed by a gradual decline over the next decade, with fewer than six thousand deaths projected for 2030–2034. This estimate is slightly lower than those from previous studies. The age-specific predictions align with the pattern of asbestos consumption and the related PM risk by birth cohort, particularly the 1940–49 cohort at the national level.

Our study is the first to apply corrections to ICD-9 frequencies based on the observed proportions of PM among all pleural tumors, using year-specific data and multiple cause of death records. Marinaccio and colleagues (see Table 1) applied a fixed correction factor of 0.73—the proportion of mesothelioma among all pleural tumors—to correct uniformly across all years, along with an additional adjustment for misdiagnosed cases, assuming a 5% annual decrease in undiagnosed mesothelioma (10, 20). In contrast, Oddone et al. (8, 9) applied a correction to data from 1970 to 2002, based on the proportions reported by the regional mesothelioma registry of Tuscany, as proposed by Ferrante et al. (21). According to our data, this method provides a good approximation (Table 1). Over or underestimating the correction factors could have some consequences on the projections: without applying any correction factor the risk of the elder birth cohort would be overestimated and the mortality peak would be projected to occur later in the future; on the contrary, applying a stronger correction would result in a projected peak earlier in the future. Our correction is relative to the classification system (i.e., adjusting for the absence of a specific code in ICD-9), not to the accuracy of death certificates, which improved over time thanks to advances in diagnoses, but depends on a number of factors that are independent from the ICD. These improvements could potentially induce the proportion of PM out of all pleural tumors to decrease with time due to increased accuracy in diagnoses, even though the absolute number of PM increases because of higher awareness. We found a steeper decrease in the number of cases in the periods following the 2020–2024 peak, compared to forecasts made by other authors. Of course, we have observed data up to 2019, at least 5 years more than the previous estimates, which provided a more accurate estimation of the peak of the epidemic curve.

Age-standardised rates peaked 10–20 years earlier. This is because the Italian population is an aged population with a reversed age pyramid. The Italian population structure shows a narrower base (of younger individuals) than the top (of older individuals). The high number of pleural mesothelioma deaths expressed by the older age group has a lesser impact after age-standardisation using the World Standard Population age structure.

Our projections provide new insights into age-specific mortality. Notably, the peak has already been reached by most age classes, up to 74 years old. The age class 75+ will instead begin the decreasing trend only after 2020, as it still includes people born in the 1940s. This underscores that cohorts born after the peak of asbestos consumption in Italy are less affected than earlier cohorts, who are now reaching the oldest age groups.

Predictions are slightly different for males and females. On absolute scale, the observed M/F ratio is between 1.7 to 2.7, females showing a decrease earlier than males. The age-specific predictions pattern by regions is consistent with known occupational and environmental asbestos exposure. Of note, among females, environmental asbestos exposure includes second-hand or paraoccupational exposure.

The rate at which mesothelioma mortality declines after its peak remains uncertain. While some countries report a rapid decrease in the number of cases, others suggest a slower decline, attributed to the long latency of mesothelioma (22–25). Our results are more consistent with the latter scenario, as they indicate a gradual decline in case numbers rather than an abrupt drop (a 5% decrease from 2020 to 2024, followed by a 10% decrease from 2030 to 2034). This pattern supports the hypothesis of a prolonged environmental exposure due to residual asbestos.

Asbestos consumption, as reported in (10), peaked in 1970–1980 and decreased progressively until the 1992 ban. Therefore, there was not an abrupt discontinuity in exposure to asbestos, and the predicted pattern reflects the smoothed decrease in exposure. Estimating the time lag between asbestos prohibition and effects on the population health is certainly not an easy task. At present, more than 60 countries have implemented a national ban on asbestos production and use. In some countries, with a huge level of international trade, asbestos is not banned and is still used massively (e.g., Russia, China, India). Furthermore, data on ARDs incidence or mortality are generally not available for these countries or clearly inadequate, according to the level of asbestos consumption (26). The absolute first countries to introduce restrictions (already in the 1970s) were the Nordic European ones. Among these, Denmark (which banned all asbestos use in 1980) predicted a peak in mortality of mesothelioma in 2015 (27). Just a few years later, in 1986, Great Britain also prohibited asbestos production, and predicted mortality from asbestos-related diseases (mesothelioma mortality) is expected to peak around 2016–2017; similarly, the Netherlands (first of a series of bans in 1991) projected a peak of PM deaths in 2017–2018 (22, 23, 28, 29). Spain, which did not introduce its first asbestos regulations until 2002, projected a continued increase in pleural cancer mortality at least through 2016; however, no subsequent decline has been estimated yet (30). France (first ban introduced in 1996) predicted two scenarios: an optimistic scenario with a peak in male mesothelioma mortality in 2030, and a more pessimistic one extending the peak to 2040 (31). In contrast, Greece, where asbestos use was relatively limited compared to other European countries, implemented a ban only in 2005, yet projected a peak in mesothelioma mortality as early as 2011 (32). Slovenia banned asbestos-cement products in 1996 and predicted the peak of mortality in 2020–2025 (33). A Europe-wide projection made before the complete asbestos ban—including chrysotile—in all EU member states in 2005, estimated the peak of mesothelioma mortality to occur around 2018 (34). Outside of Europe, predictions were made for Australia (ban in 2003, and mesothelioma peak in 2030); Brazil (partial ban on about 70% of production in 2000–2001, projected mesothelioma peak in 2021–2026); South Korea (ban in 2009, with a male mesothelioma peak projected for 2029–2033 and a female peak for 2024–2028) (35–37). Concerning the North American countries, as already mentioned, the USA removed a previously implemented national ban on asbestos (1989) and started to observe a decrease in incidence as a result of the earlier policy; a series of local restrictions are now in place (38). On the other side, Canada, which alone contributed to around 50% of global asbestos production, introduced a national ban only in 2018 (although production had already decreased) and predicts a peak for males in 2020 and females in 2030 (39). A recent study conducted using GBD 2021 and GLOBOCAN 2022 databases presented global projections of mesothelioma up to 2050, obtained by applying ML models. The results show a remarkable difference in projections between low and high SDI countries, with the latter exhibiting higher death rates (40). Overall, rates are predicted to remain stable in both sexes until the end of the prediction period. In light of these considerations, the results presented in this paper are in agreement in placing the peak of absolute PM mortality around 30 years after the introduction of asbestos bans. Nonetheless, when making international comparisons, caution should always be exercised to account for the fact that differences in diagnosis and case registration may exist among countries.

Our study is the first to make country-wide predictions at the regional level; previously, only some region-specific projections were available, primarily for the northern regions. A study for Veneto region, which used mesothelioma mortality up to 2010 (13), reported mesothelioma incidence predictions that reached a peak in the years 2010–2014 for the age group 70–74. Predictions for Lombardy were also made regarding the incidence of mesothelioma, predicting a peak in 2019 with 417 annual cases, similarly to our results (14). A study analyzing and projecting mortality for Sicily predicted a peak in male mortality for PM in 2021 (15). The same study, along with several other articles, also presents specific projections for known contaminated sites, including the Sicilian ones (Augusta-Priolo, Milazzo, and Gela); the cohort of Breda factory workers in Pistoia (Tuscany); and the city of Casale Monferrato (Piedmont), hosting the largest asbestos-cement factory in Italy (15, 41–43). Our figures reflect the history of asbestos consumption and occupational exposures within Italy: among males, particularly in shipyards in Friuli Venezia Giulia (highest risk for birth cohort 1940–49) and Liguria (historically the oldest, the highest risk for the birth cohort 1930–39), as well as in the mine and asbestos-related industries in Lombardy and Piedmont, and, among females, asbestos-related industries and textile industries in Lombardy and Piedmont (with slightly different birth cohort profiles).

The main strengths of our analysis lie in the data that we used, which cover a wide period and, for the first time, are adjusted for the real proportion of misclassified pleural tumors. We applied the proportion observed in 1995, the year the multiple cause of death register was implemented, to previous years. There is indeed no reason to believe that the proportion of mesothelioma out of all pleural cancers would be significantly different over the years between 1980 and 1994.

Among study limitations, we can include the use of aggregate data rather than individual-level exposure histories. In addition, potential prognosis improvements were not explicitly modelled. Nonetheless, the BAPC model also considers a period effect, not only a birth cohort one. Therefore, albeit unlikely, improvements in prognosis could be caught by a calendar period effect, although this is not testable due to identifiability issues. Projections were made separately for males and females. Further stratifications (e.g., based on occupation or socioeconomic factors) were not made due to the lack of available information. Moreover, our analyses miss information on environmental asbestos exposure, still ongoing in our country, particularly due to the asbestos present in existing buildings. We still do not have enough evidence on this specific type of exposure from cancer registries, but it would be of fundamental importance for future work.

Our results update and refine the existing literature on the topic, providing valuable insights into regional trends. The estimated number of mesothelioma deaths in the next years, by regional areas, could contribute to planning public health actions, including the required economic resources, to ensure adequate assistance to the victims and their families. Moreover, the age-group observed and predicted rates show that the peak of PM has already been reached in almost all classes. These findings are a fundamental tool to demonstrate the association between asbestos prohibition and the reduction of ARDs mortality.

## Data Availability

The data analyzed in this study is subject to the following licenses/restrictions: the data that support the findings of this study are available from Italian National Institute of Health, but restrictions apply to the availability of these data, which were used under license for the current study, and so are not publicly available. Data are however available from the authors upon reasonable request and with permission of Istituto Superiore di Sanità. Requests to access these datasets should be directed to Giada Minelli, giada.minelli@iss.it.
