# Supplementary material for: Projecting future mortality risk of pleural mesothelioma in Italy (2020–2034): Bayesian age–period–cohort analysis over 40 years of death registration
Source: Front Public Health. 2026 Jan 16;13:1741506. doi: 10.3389/fpubh.2025.1741506 (PMC12856937; doi:10.3389/fpubh.2025.1741506)

Supplementary Material

# Supplementary Figures and Tables

**Supplementary Table 1**: Lexis Diagram showing age classes, calendar period and birth cohorts. Highlighted in grey are the birth cohorts used for the analyses.

**Supplementary Table 2**: Observed and Predicted M/F Ratio. **Italy, 1980-2034.**

| **Calendar Period** | **Observed M/F Ratio** | **Predicted M/F Ratio** |
| --- | --- | --- |
| 1980 - 1984 | 1.680 | 1.617 |
| 1985 - 1989 | 1.706 | 1.717 |
| 1990 - 1994 | 1.782 | 1.852 |
| 1995 - 1999 | 2.011 | 2.037 |
| 2000 - 2004 | 2.222 | 2.245 |
| 2005 - 2009 | 2.570 | 2.473 |
| 2010 - 2014 | 2.663 | 2.625 |
| 2015 - 2019 | 2.661 | 2.707 |
| 2020 - 2024 |  | 2.757 |
| 2025 - 2029 |  | 2.784 |
| 2030 - 2034 |  | 2.724 |

**Supplementary Table 3**: CRPS calibration test results. Z statistic (p-value).

| **Age group** | **Males** | **Females** |
| --- | --- | --- |
| 45-49 | 0.466 (0.642) | -0.220 (0.826) |
| 50-54 | -0.925 (0.355) | -0.903 (0.366) |
| 55-59 | -0.969 (0.333) | 0.705 (0.481) |
| 60-64 | -0.511 (0.610) | -1.743 (0.081) |
| 65-69 | -0.866 (0.386) | -1.293 (0.196) |
| 70-74 | -1.136 (0.256) | -1.498 (0.134) |
| 75-79 | -1.186 (0.236) | 0.757 (0.449) |
| 80-84 | -0.776 (0.438) | -1.091 (0.275) |
| 85+ | -0.510 (0.610) | 0.317 (0.751) |

## Supplementary Figures


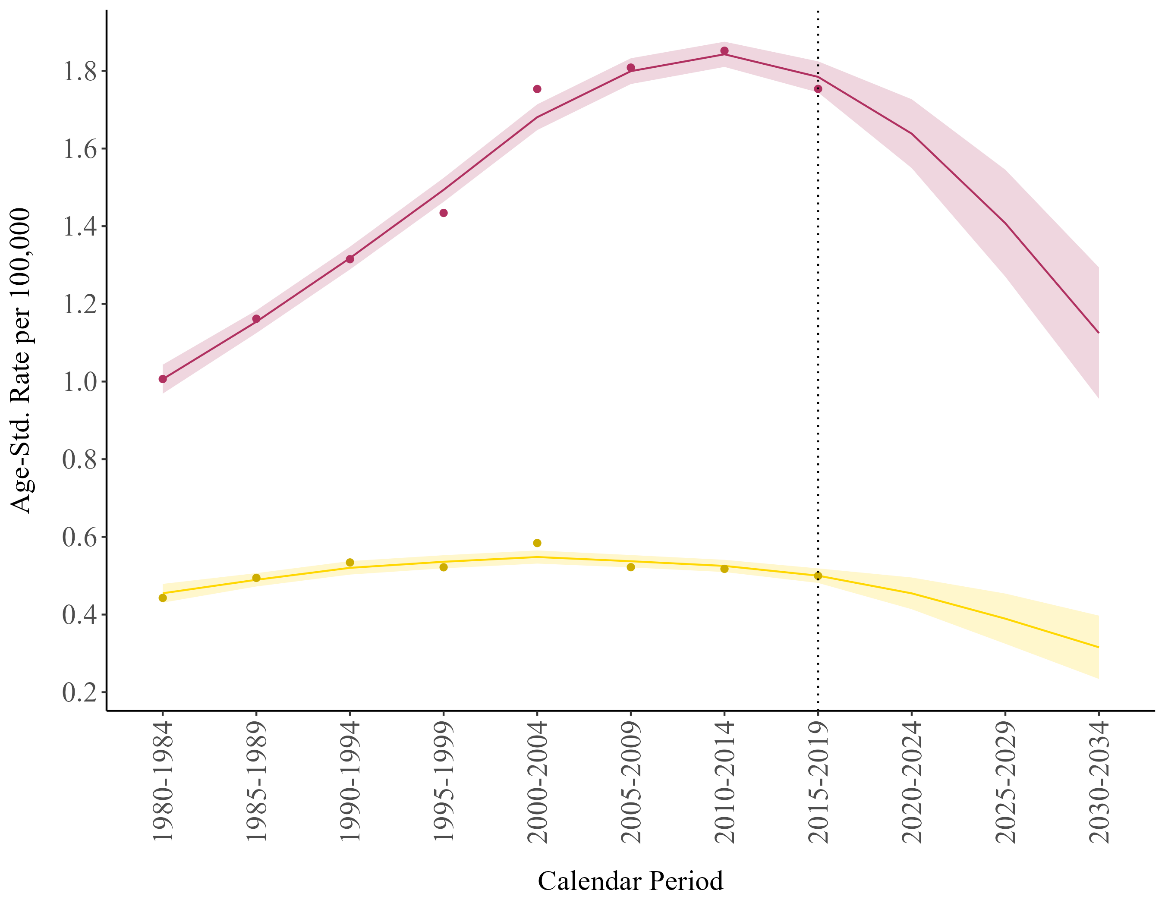
**Supplementary Figure 1:** Projected and observed (dots) age-standardized mortality rates (using the 2013 revision of the European Standard population), for males (dark red) and females (sand), with 90% credibility intervals. **Italy, 1980-2034.**


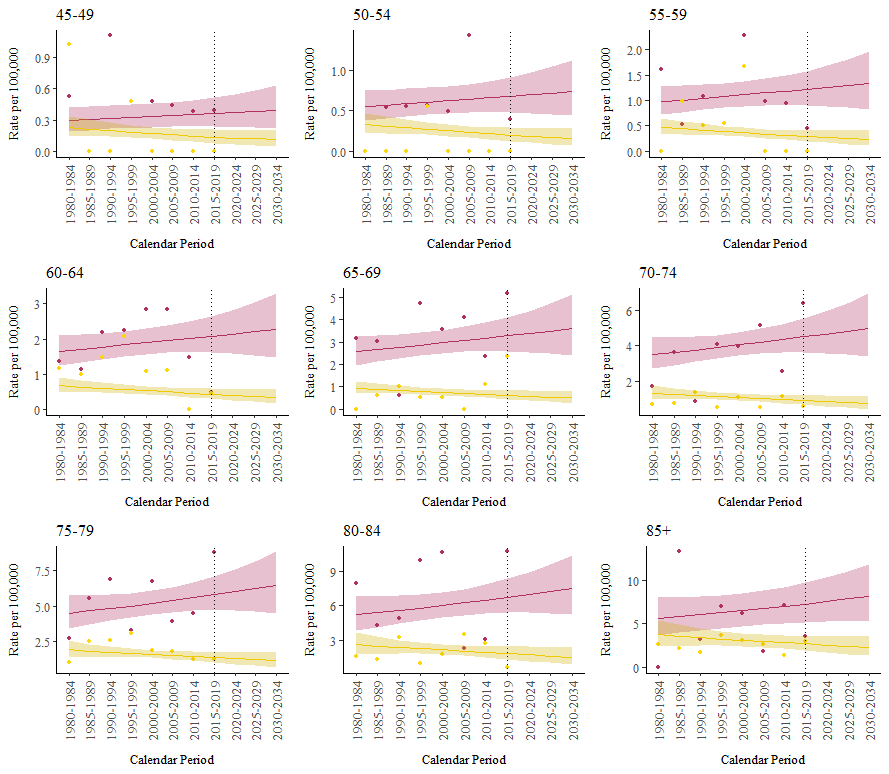
Supplementary Figure 2: Projected and observed age-specific mortality rates (per 100,000) by calendar period, for males (Panel A) and females (Panel B) with 90% credibility intervals. Abruzzo, 1980-2034.


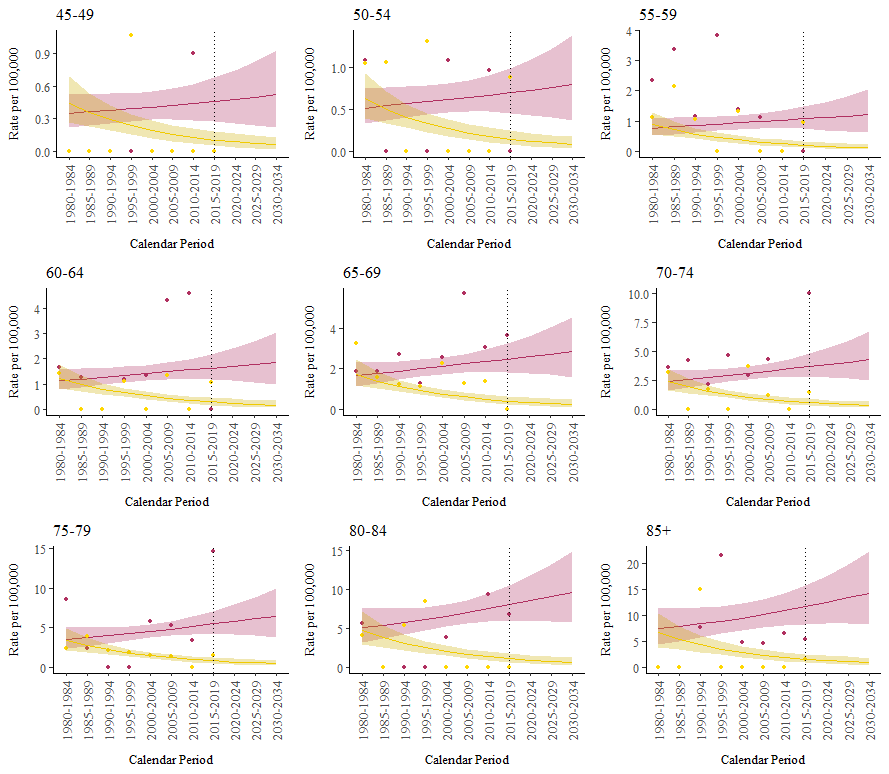
 Supplementary Figure 3: Projected and observed age-specific mortality rates (per 100,000) by calendar period, for males (Panel A) and females (Panel B) with 90% credibility intervals. Basilicata, 1980-2034.


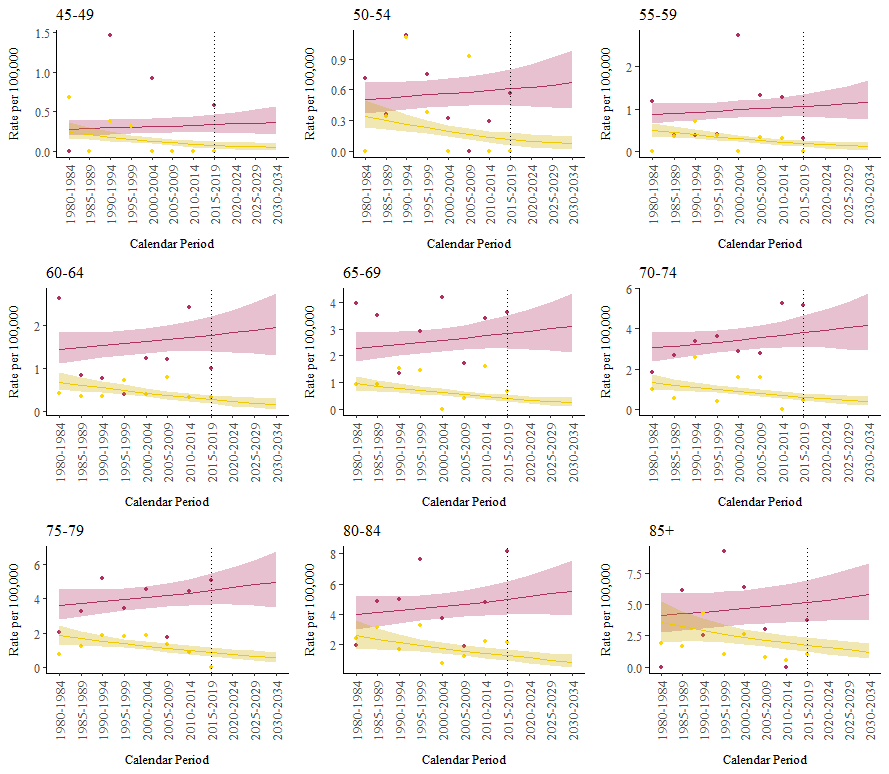
Supplementary Figure 4: Projected and observed age-specific mortality rates (per 100,000) by calendar period, for males (Panel A) and females (Panel B) with 90% credibility intervals. Calabria, 1980-2034.


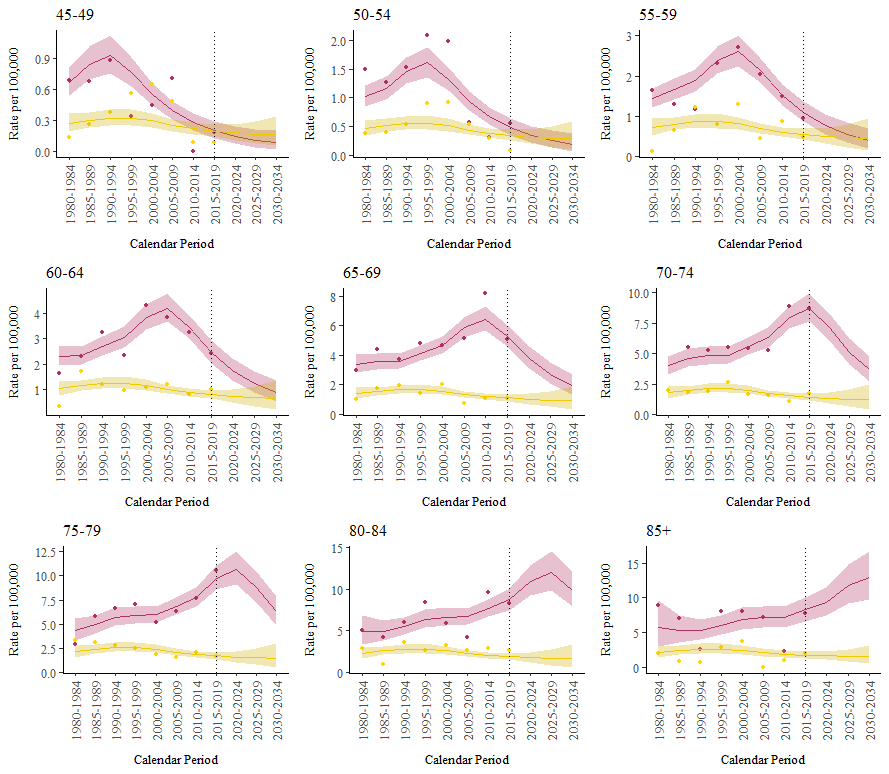
**Supplementary Figure 5**: Projected and observed age-specific mortality rates (per 100,000) by calendar period, for males (Panel A) and females (Panel B) with 90% credibility intervals. Campania, 1980-2034.


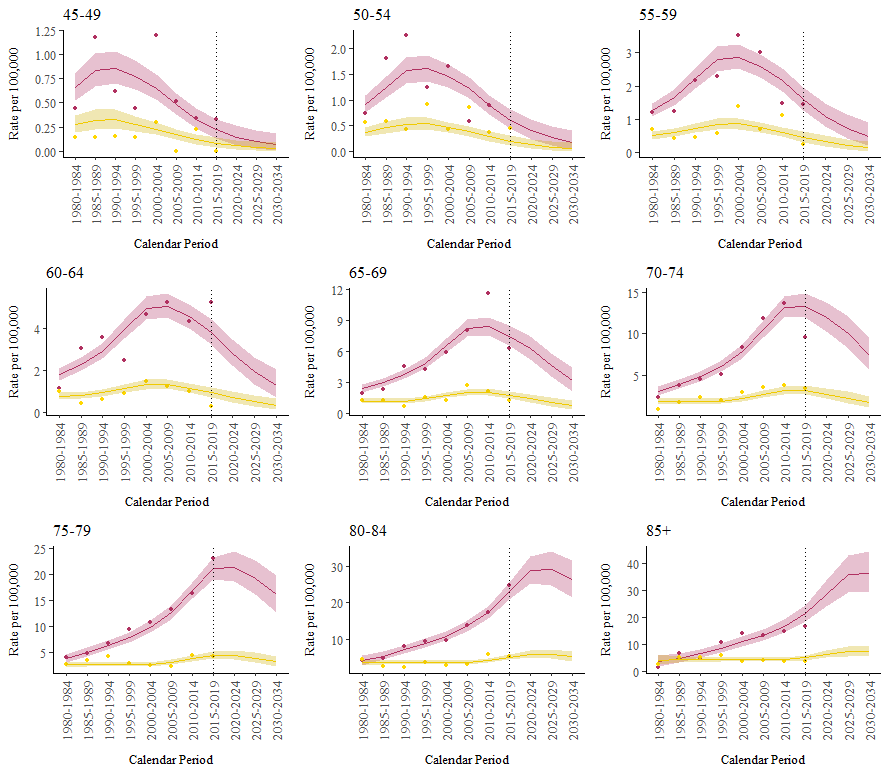
Supplementary Figure 6: Projected and observed age-specific mortality rates (per 100,000) by calendar period, for males (Panel A) and females (Panel B) with 90% credibility intervals. Emilia-Romagna, 1980-2034.


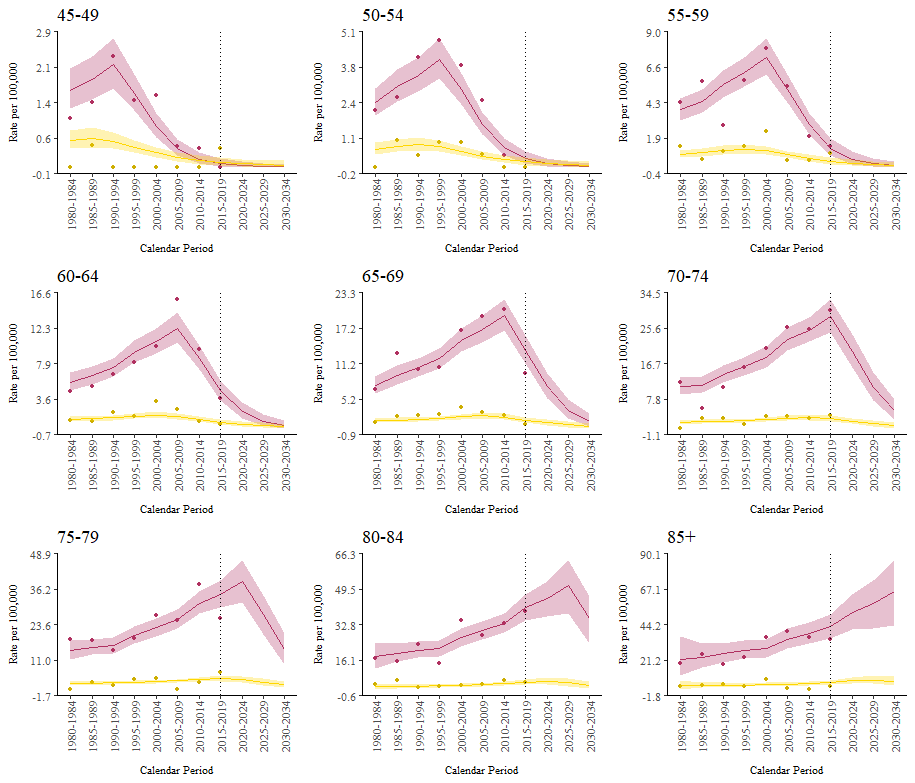
**Supplementary Figure 7**: Projected and observed age-specific mortality rates (per 100,000) by calendar period, for males (Panel A) and females (Panel B) with 90% credibility intervals. Friuli-Venezia Giulia, 1980-2034.

**Supplementary Figure 8**: Projected and observed age-specific mortality rates (per 100,000) by calendar period, for males (Panel A) and females (Panel B) with 90% credibility intervals. Lazio, 1980-2034.


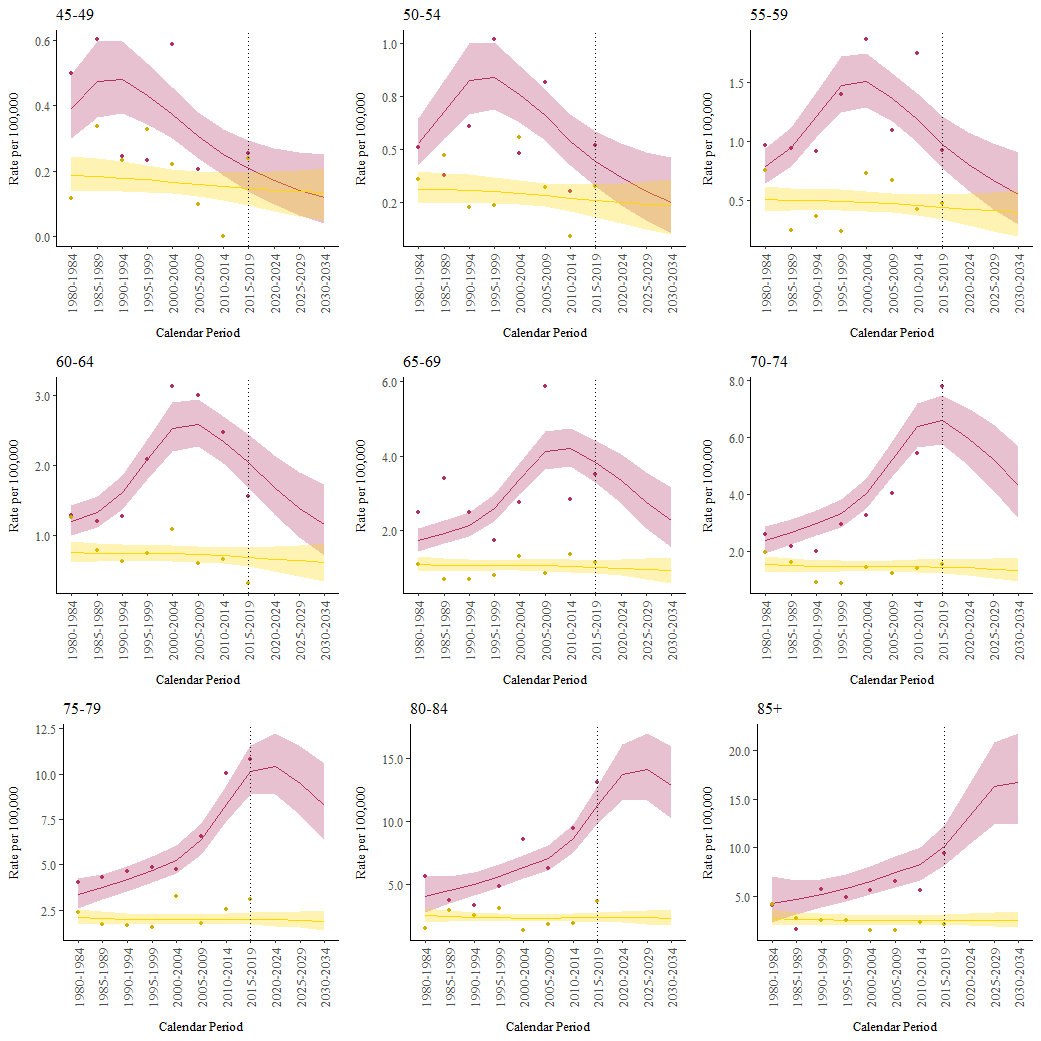


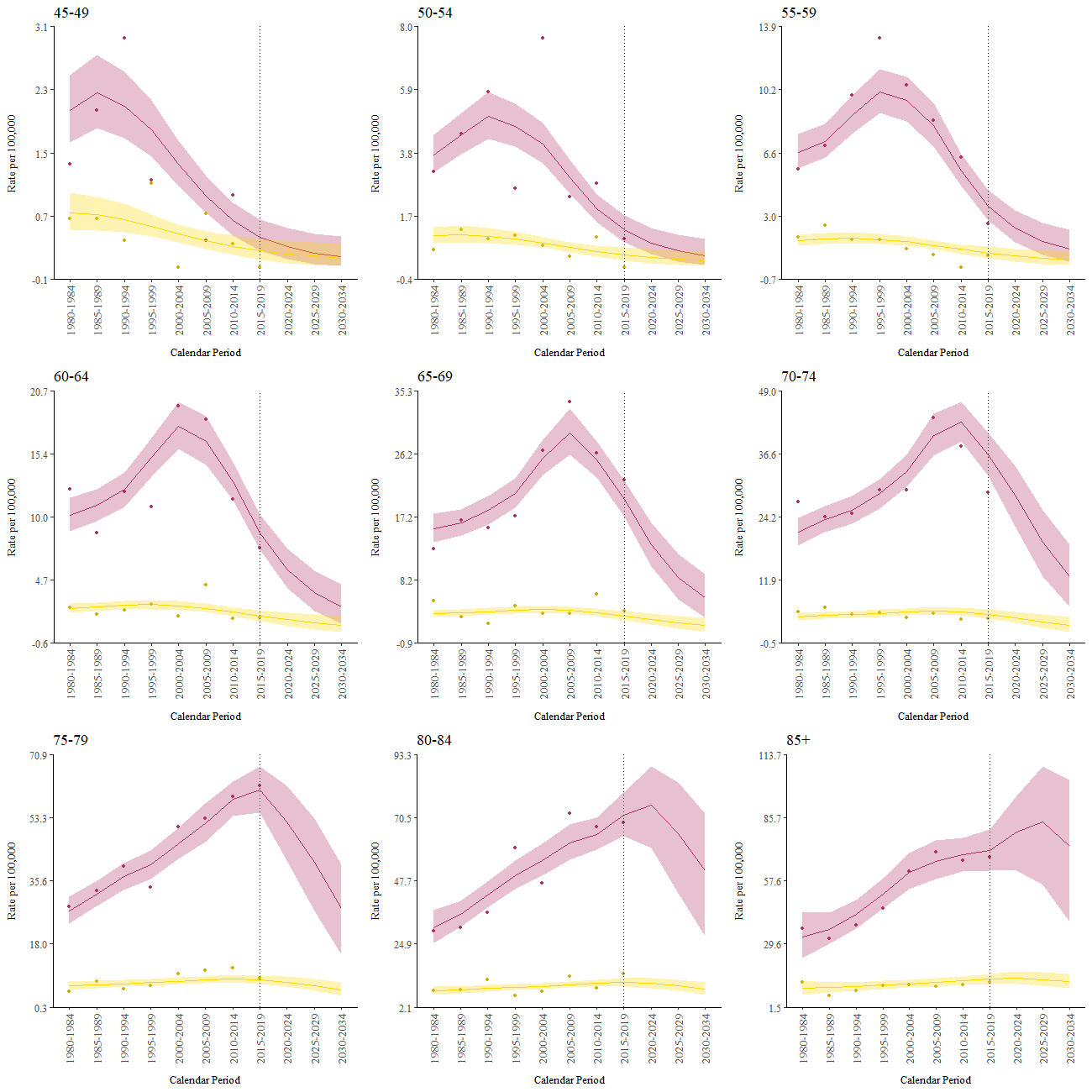
Supplementary Figure 9: Projected and observed age-specific mortality rates (per 100,000) by calendar period, for males (Panel A) and females (Panel B) with 90% credibility intervals. Liguria, 1980-2034.


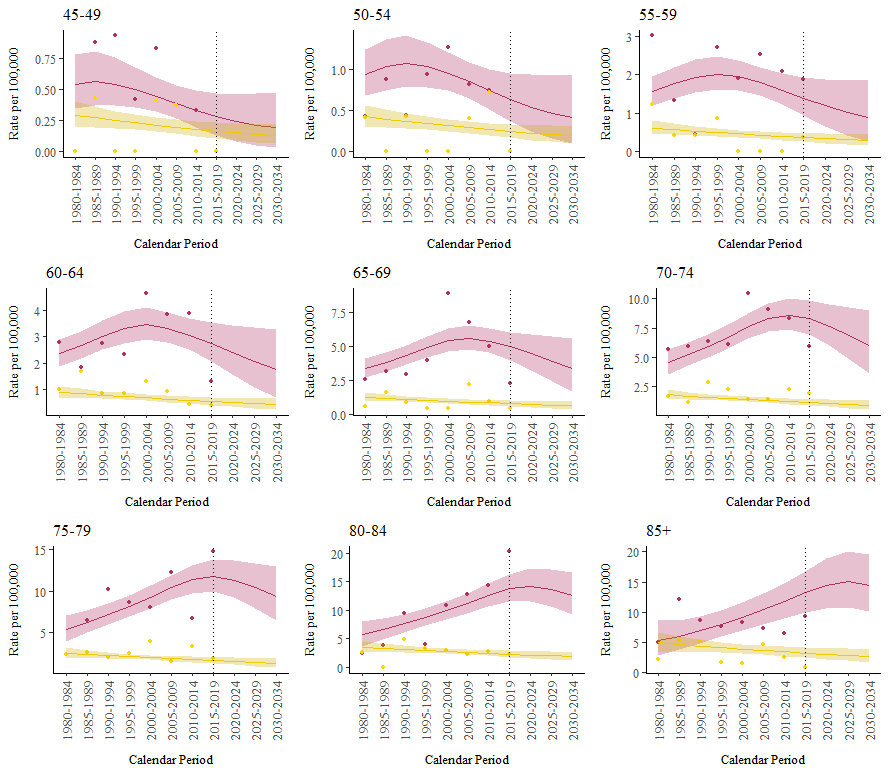
Supplementary Figure 10: Projected and observed age-specific mortality rates (per 100,000) by calendar period, for males (Panel A) and females (Panel B) with 90% credibility intervals. Marche, 1980-2034.

**
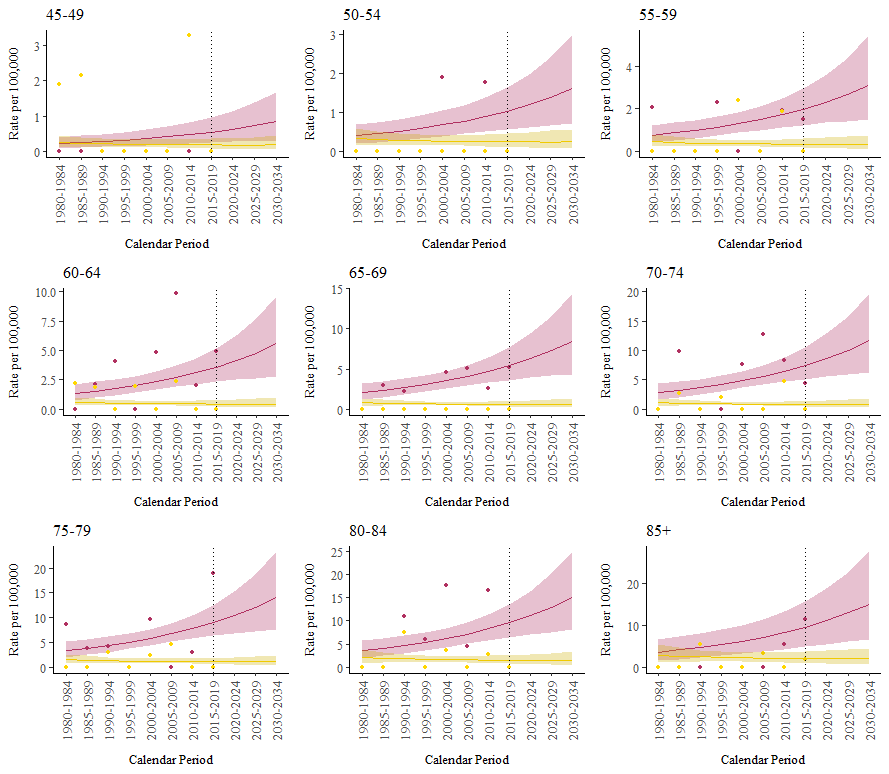
Supplementary Figure 11:** Projected and observed age-specific mortality rates (per 100,000) by calendar period, for males (Panel A) and females (Panel B) with 90% credibility intervals. Molise, 1980-2034

**Supplementary Figure 12:** Projected and observed age-specific mortality rates (per 100,000) by calendar period, for males (Panel A) and females (Panel B) with 90% credibility intervals. Apulia, 1980-2034.


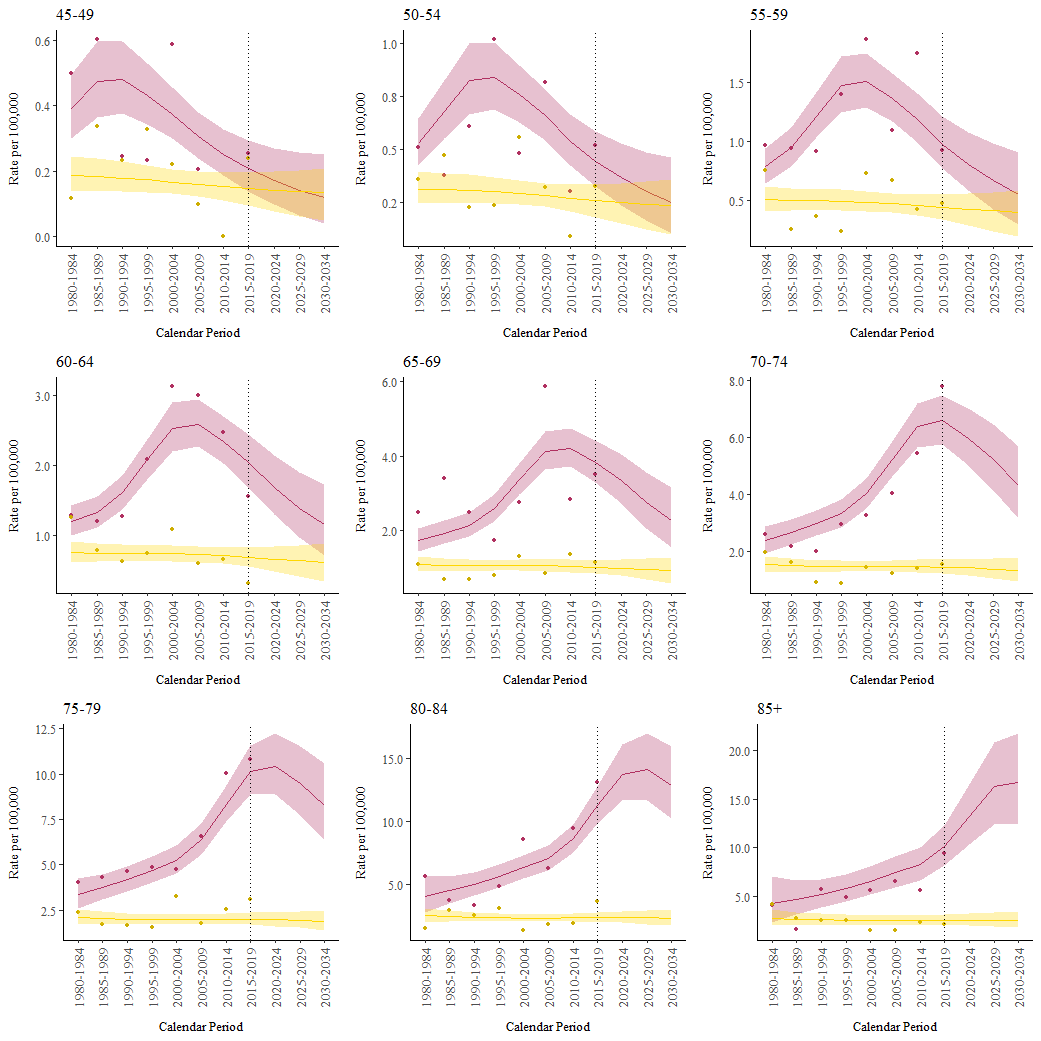


**Supplementary Figure 13:** Projected and observed age-specific mortality rates (per 100,000) by calendar period, for males (Panel A) and females (Panel B) with 90% credibility intervals. Sardinia, 1980-2034.


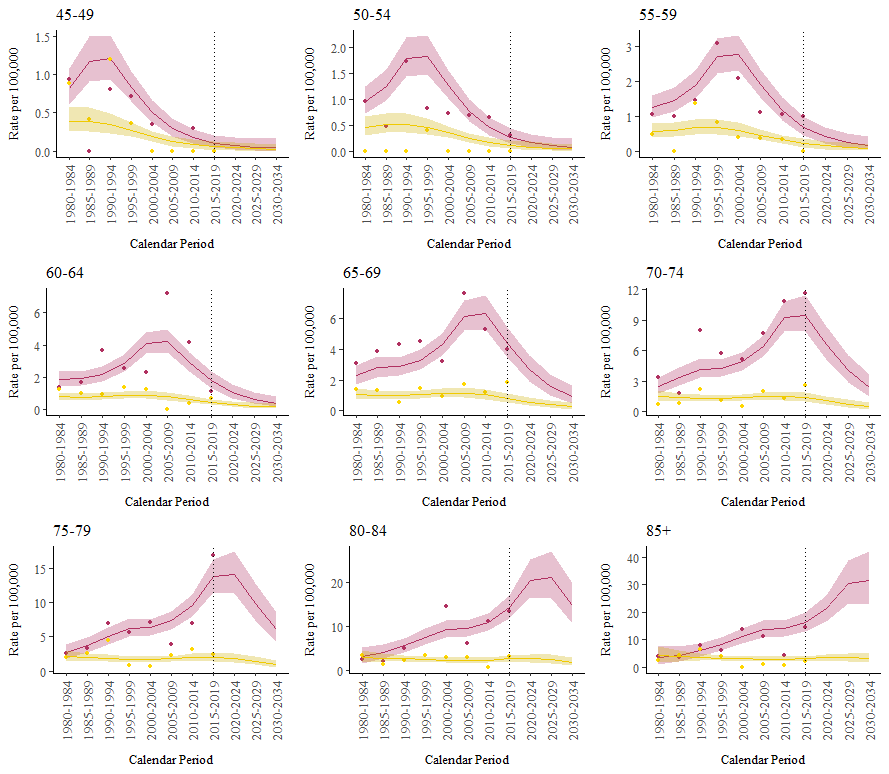


**Supplementary Figure 14:** Projected and observed age-specific mortality rates (per 100,000) by calendar period, for males (Panel A) and females (Panel B) with 90% credibility intervals. Sicily, 1980-2034.


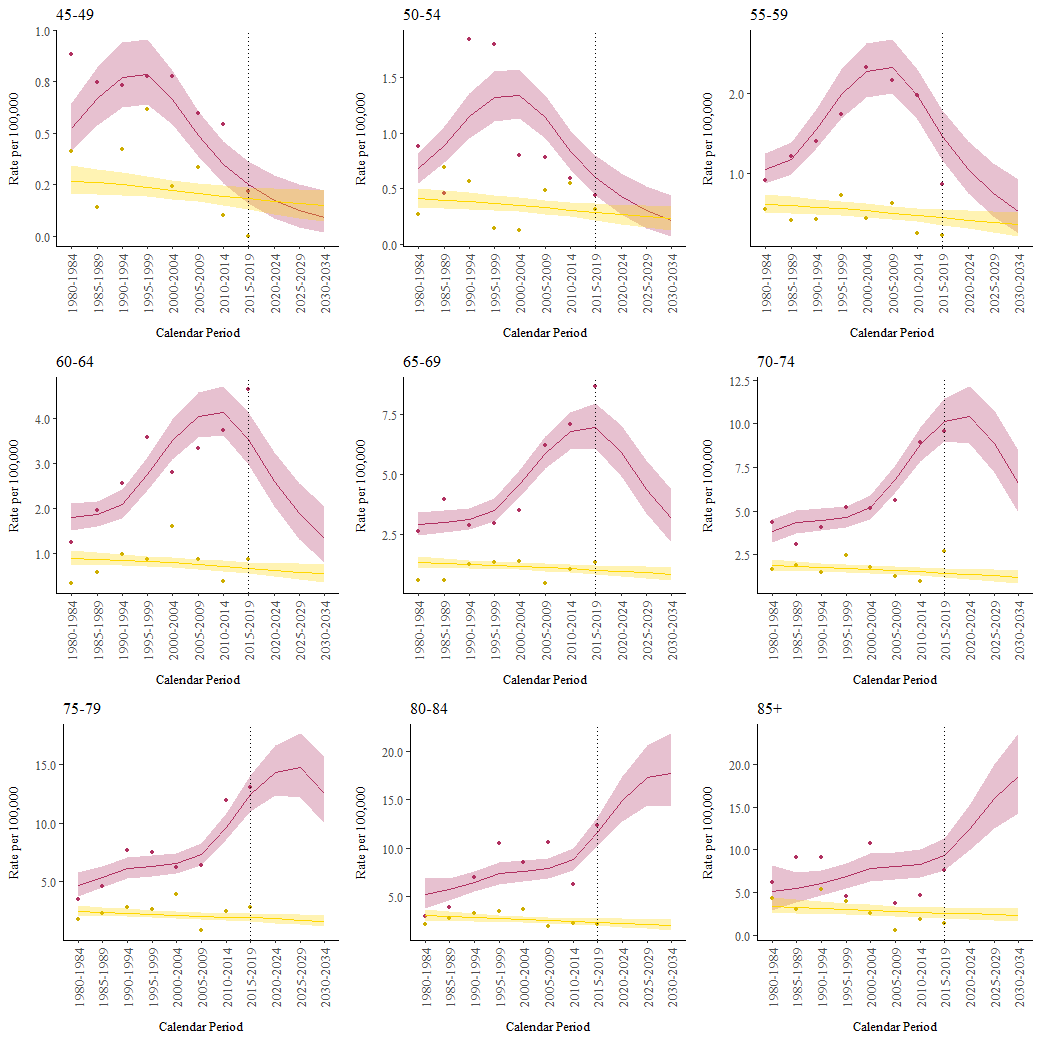


**Supplementary Figure 15:** Projected and observed age-specific mortality rates (per 100,000) by calendar period, for males (Panel A) and females (Panel B) with 90% credibility intervals. Toscana, 1980-2034.


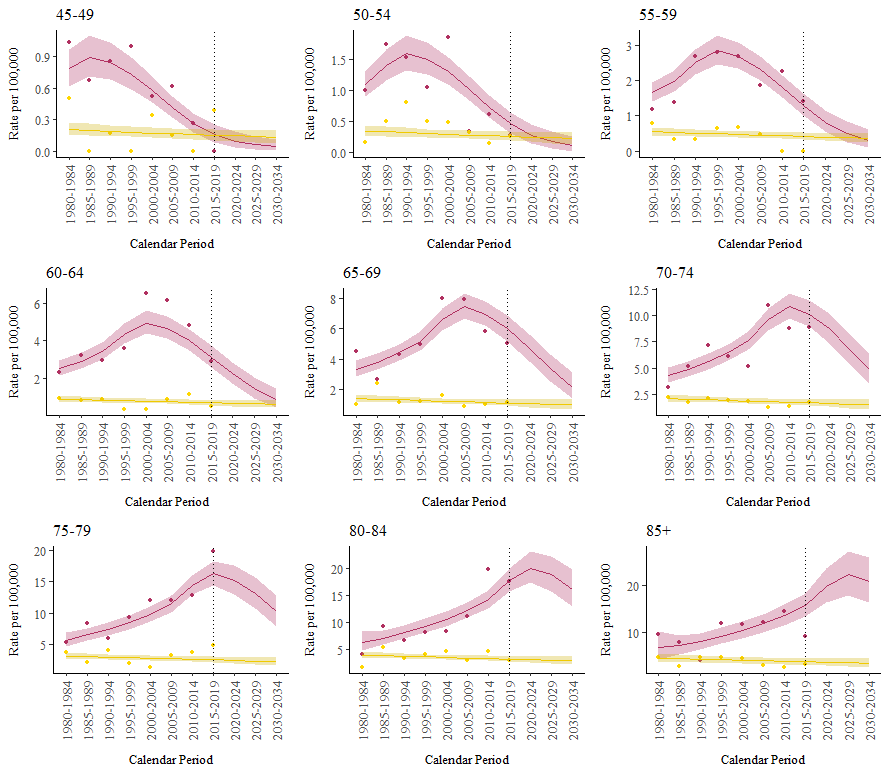


**Supplementary Figure 16:** Projected and observed age-specific mortality rates (per 100,000) by calendar period, for males (Panel A) and females (Panel B) with 90% credibility intervals. Trentino-Alto Adige, 1980-2034.


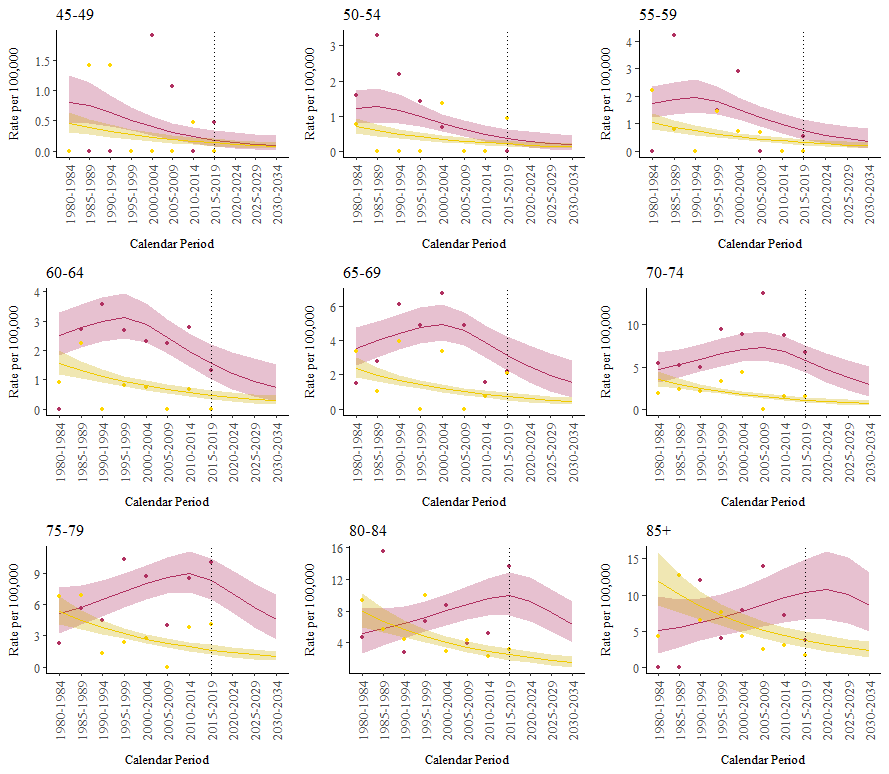


**Supplementary Figure 17:** Projected and observed age-specific mortality rates (per 100,000) by calendar period, for males (Panel A) and females (Panel B) with 90% credibility intervals. Umbria, 1980-2034.


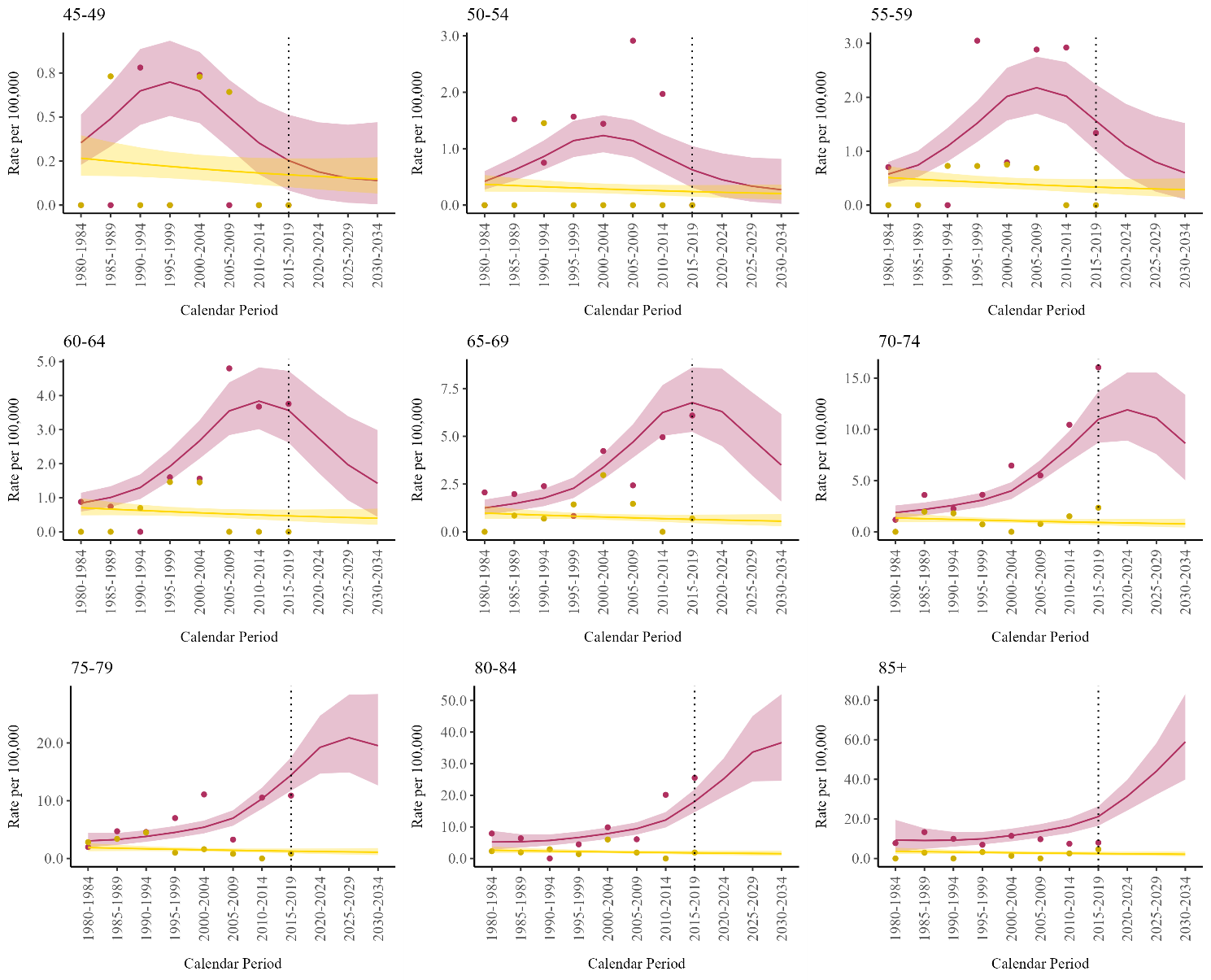


**Supplementary Figure 18:** Projected and observed age-specific mortality rates (per 100,000) by calendar period, for males (Panel A) and females (Panel B) with 90% credibility intervals. Valle d’Aosta, 1980-2034.


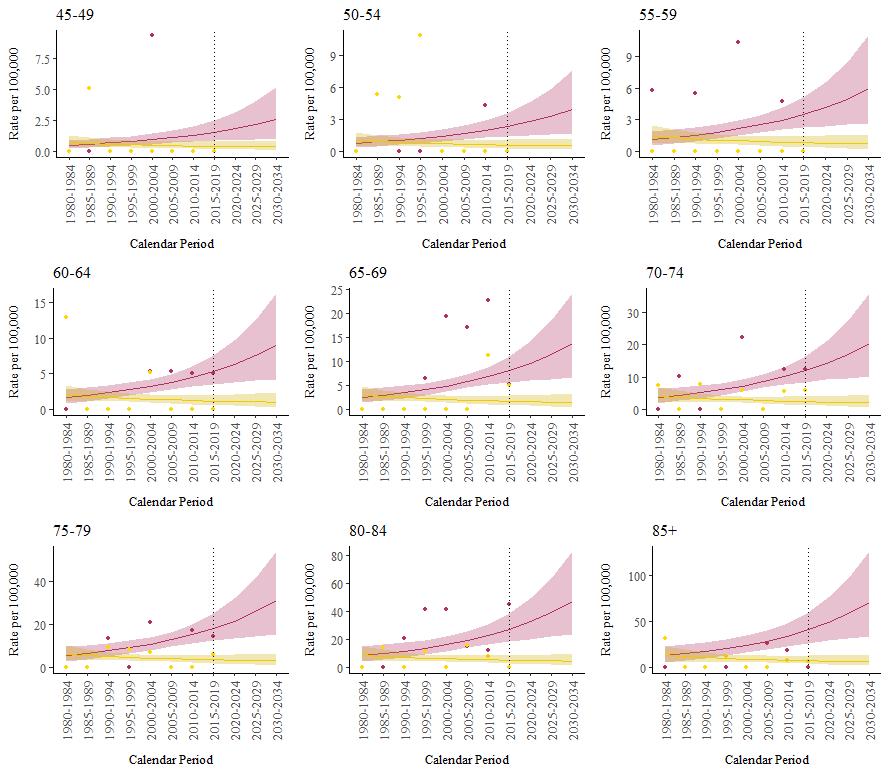


**Supplementary Figure 19:** Projected and observed age-specific mortality rates (per 100,000) by calendar period, for males (Panel A) and females (Panel B) with 90% credibility intervals. Veneto, 1980-2034.


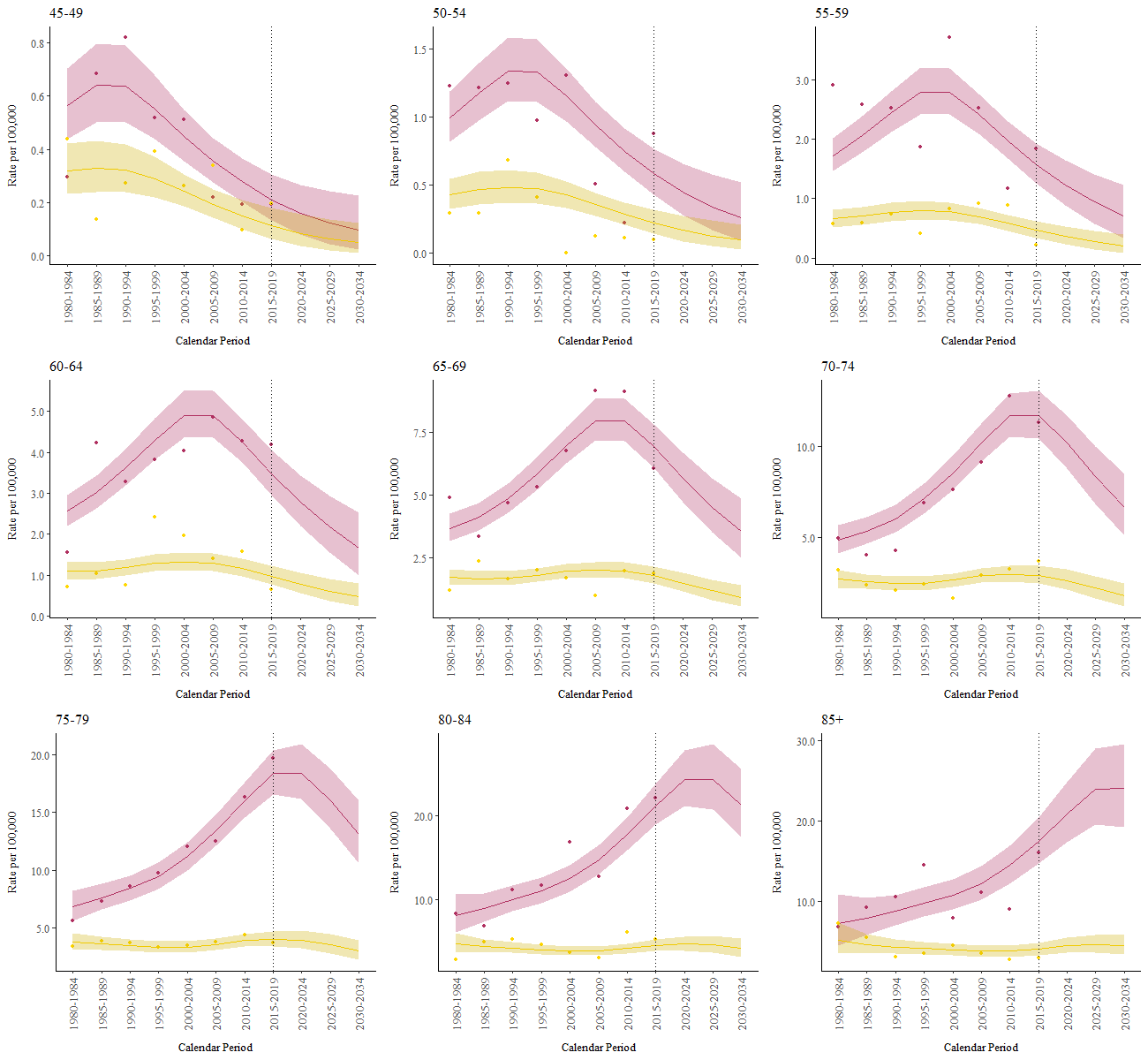

Supplement: Supplementary file 1 [file Data_Sheet_1.zip › Supplementary_Material.docx]
